# Preliminary Assessment of Pisotriquetral Joint Kinematics Following Transverse Carpal Ligament Release: A Cadaveric Pilot Study

**DOI:** 10.7759/cureus.101067

**Published:** 2026-01-08

**Authors:** Raphael Israeli, Chen Steinmetz, Gil Gannot, Amir Oron

**Affiliations:** 1 Department of Orthopedic Surgery, Kaplan Medical Center, Rehovot, ISR; 2 Faculty of Medicine, The Hebrew University of Jerusalem, Jerusalem, ISR

**Keywords:** cadaveric study, carpal tunnel release, pisotriquetral arthritis, pisotriquetral joint, transverse carpal ligament, ulnar-sided wrist pain, wrist biomechanics

## Abstract

Introduction

Transverse carpal ligament (TCL) release is the standard treatment for carpal tunnel syndrome; however, its potential to alter carpal biomechanics is unclear. Specifically, it has been hypothesized that TCL transection may destabilize the pisotriquetral (PT) joint or unmask latent arthritis. This pilot study investigated the immediate kinematic effects of TCL transection on the PT joint in an embalmed cadaveric model.

Methods

Six embalmed cadaveric wrists were mounted on a custom stabilization apparatus. Colored markers were affixed to the ulna, pisiform, and fourth metacarpal bones to track the motion vectors. Wrist kinematics were recorded via video analysis before and after complete TCL transection under simulated maximal flexor carpi ulnaris (FCU) contraction. Changes in wrist flexion, extension, and relative PT angle were quantified.

Results

In this cohort, TCL release did not result in statistically significant changes in kinematic parameters. The mean difference in wrist extension was -2.83° (95% CI: -9.72° to 4.05°; p = 0.34). The mean difference in wrist flexion was -2.84° (95% CI: -10.57° to 4.88°; p = 0.39). The PT angular relationship remained consistent pre- and post-release (mean difference: -0.01°; 95% CI: -2.84° to 2.82°; p = 0.99).

Conclusions

Within the limitations of a small sample size and embalmed tissue model, complete TCL release did not produce gross kinematic alterations in the PT joint. While these preliminary findings suggest that the intrinsic ligamentous stability of the pisiform is maintained post-release, larger studies utilizing fresh-frozen specimens are required to rule out subtle instability patterns that may contribute to postoperative ulnar-sided wrist pain.

## Introduction

The pisotriquetral (PT) joint serves as a critical biomechanical fulcrum for the flexor carpi ulnaris (FCU) tendon, facilitating effective force transmission during wrist flexion and ulnar deviation [1,2]. The FCU is essential for forceful hand movements, rendering the functional integrity of the PT joint significant [2]. Despite its importance, PT joint pathology is often overlooked [3]. Primary PT arthritis is rare, accounting for approximately 2.3% of cases, whereas secondary PT arthritis is far more common, comprising nearly half of all PT joint pathologies, typically caused by trauma or instability [4,5].

A persistent clinical challenge following carpal tunnel release (CTR) is the development or persistence of ulnar-sided wrist pain, which may arise from altered carpal biomechanics or secondary joint pathologies [6,7]. One prevailing biomechanical hypothesis suggests that transection of the transverse carpal ligament (TCL) disrupts the stabilizing soft tissue envelope of the carpus, as the TCL maintains essential tension in the carpal arch [8,9]. The loss of this tethering effect can alter pisiform tracking, potentially inducing dynamic instability or unmasking latent PT arthritis [8,10]. Consequently, previous studies have emphasized the importance of preserving TCL integrity and adopting limited-release approaches during CTR to maintain ulnar and carpal stability [11,12].

Previous research on the biomechanics of the pisiform has produced conflicting evidence regarding its contribution to global wrist function. While some studies indicate that pisiform excision has minimal impact on wrist biomechanics [13-15], others suggest that the pisiform is integral to wrist extension and stability [16]. Given the inconsistency of these data, further investigation is necessary to determine whether the isolated release of the TCL, with the pisiform remaining in place, significantly affects PT joint kinematics.

To address this gap, we conducted a cadaveric pilot study to evaluate PT joint biomechanics before and after TCL transection. We hypothesized that complete transection of the TCL would lead to measurable alterations in pisiform tracking and PT joint angles during wrist motion. The primary outcome of this investigation was to quantify changes in wrist flexion, extension, and the PT angle under simulated FCU loading to determine if TCL release contributes to immediate joint instability.

## Materials and methods

Study setting and specimen selection

This multicenter pilot study was conducted at the University Anatomy Laboratories. The study cohort comprised six adult cadaveric upper extremities preserved using formaldehyde fixation. The sample size (n = 6) was determined based on the availability of specimens that met strict inclusion criteria regarding tissue quality and joint mobility. Due to institutional anonymity protocols, specific demographic data (age and sex) and medical history were unavailable. However, prior to inclusion, all specimens underwent rigorous visual and physical examinations to rule out previous surgical scars, deformities, or signs of prior trauma that could confound biomechanical results. Specimens with significant rigor, contractures, or an inability to achieve a functional arc of motion were excluded from the study. Only specimens capable of demonstrating a full range of wrist motion were included. Embalmed tissue was selected based on availability, with the acknowledgment that tissue stiffness differs from that of fresh-frozen specimens. This study utilized cadaveric specimens from the University Anatomy Laboratory. In accordance with local institutional regulations, research involving cadaveric specimens is exempt from Helsinki Committee approval.

Anatomical preparation and stabilization

**Figure 1:**
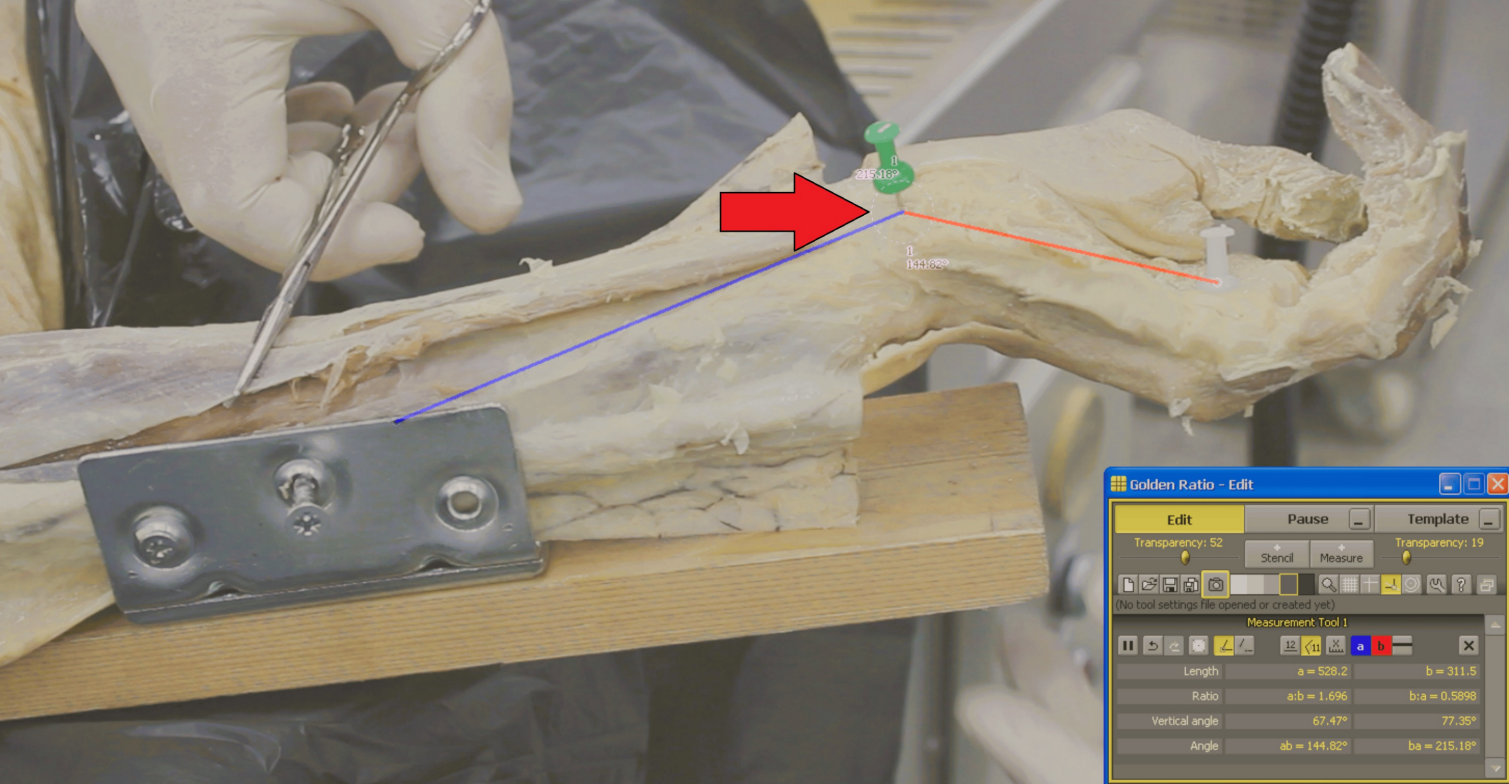
Baseline Kinematic Analysis in the Resting Position Vector analysis of the intact wrist prior to TCL release. The angle is defined by three anatomical landmarks: the proximal ulnar marker (10 cm proximal to the joint), the pisiform centroid, and the distal 4th metacarpal marker (7 cm distal to the joint). No load is applied to the FCU. TCL, transverse carpal ligament; FCU, flexor carpi ulnaris

Superficial dissection was performed on each forearm to expose the FCU muscle and tendon unit, ensuring that the tendon was free from adhesions to allow for excursion testing. To isolate wrist motion and neutralize forearm rotation during the kinematic evaluation, the forearm was rigidly secured to a custom stabilization apparatus using two Schanz screws driven into the ulnar shaft (Figure 1). This configuration allowed the carpus and hand to move freely while maintaining the static ulnar reference.

Kinematic marker placement

To facilitate the vector analysis of joint motion, three distinct anatomical landmarks were identified and marked with high-contrast color-coded pins. These markers were used to define the longitudinal axes of the forearm and hand relative to the pisiform. The proximal reference was placed on the ulnar shaft, exactly 10 cm proximal to the pisiform. The joint center was defined as the pisiform bone itself. The distal reference was placed on the fourth metacarpal, exactly 7 cm distal to the pisiform. These standardized distances were maintained across all specimens to ensure consistent calculations.

Motion capture and protocol

**Figure 2:**
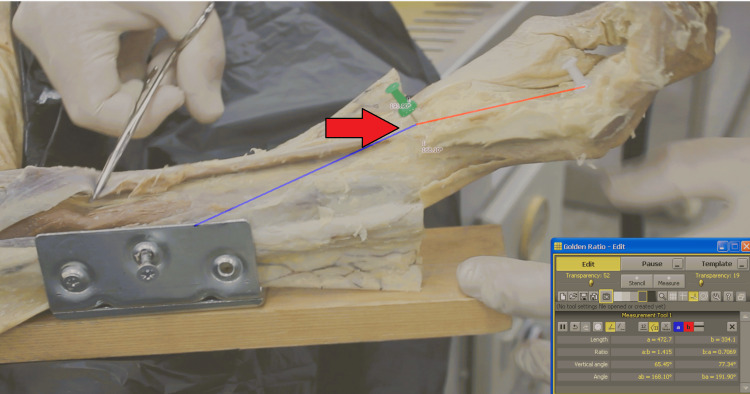
Baseline Kinematic Analysis During Simulated Muscle Loading Vector analysis of the intact wrist during simulated maximal FCU contraction. Manual traction is applied to the exposed tendon to reproduce forceful flexion prior to TCL release. TCL, transverse carpal ligament; FCU, flexor carpi ulnaris

Kinematic data were captured using a video camera mounted at a fixed distance of 50 cm from the forearm, positioned orthogonally to the plane of motion to minimize parallax error. A sequential testing protocol was used. Initially, baseline kinematic data for the intact wrist were recorded in the resting, unloaded position (Figure 1), followed by a simulated maximal FCU contraction (Figure 2). To simulate the primary stabilizing force on the PT joint, manual traction was applied to the exposed FCU tendon at the point of maximal excursion of the PT joint.

Surgical intervention

**Figure 3:**
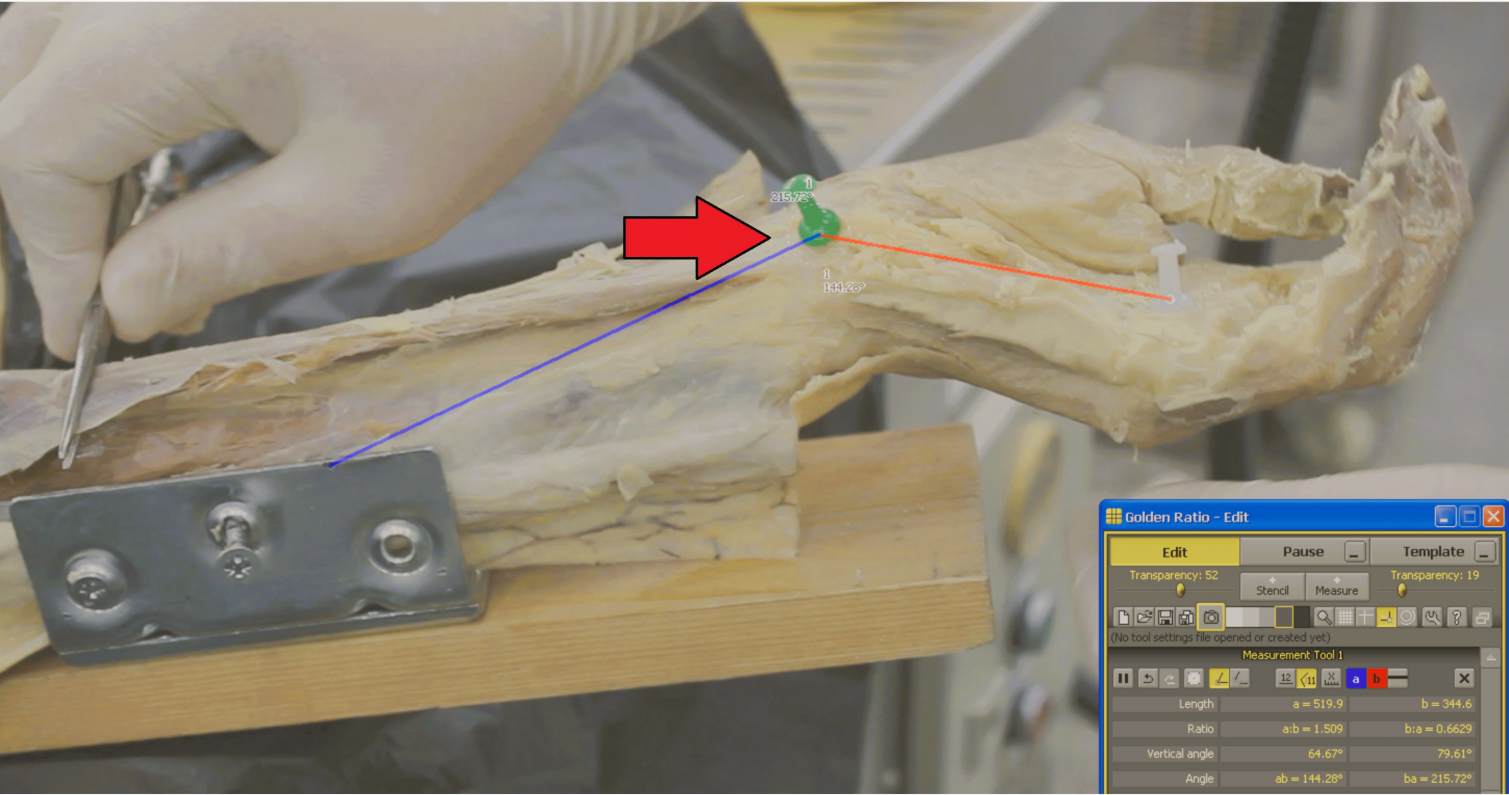
Post-release Kinematic Analysis in the Resting Position Vector analysis of the wrist following complete transection of the TCL. The image depicts the PT angle in the neutral, unloaded state. TCL, transverse carpal ligament; PT, pisotriquetral

**Figure 4:**
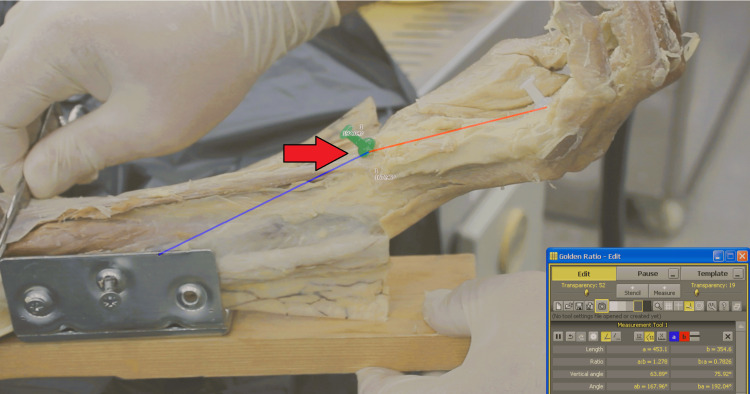
Post-release Kinematic Analysis During Simulated Muscle Loading Vector analysis of the wrist following complete transection of the TCL during simulated maximal FCU traction. TCL, transverse carpal ligament; FCU, flexor carpi ulnaris

Following the baseline measurements, a formal open CTR was performed. The TCL was completely transected under direct visualization to ensure full release of the tendon. Following the intervention, the range of motion and loading protocols were repeated using the identical apparatus and camera positioning to ensure consistency. The wrist was recorded again in the resting position (Figure 3) and under simulated FCU traction (Figure 4).

Data analysis

Video data were analyzed using Golden Ratio software, version 3.1 (Markus Welz, http://www.markuswelz.de/software2/). This platform tracked the relative vectors between the defined landmarks to quantify the PT angle, wrist flexion, and extension. Statistical analyses were performed using IBM SPSS Statistics for Windows, version 21 (released 2012; IBM Corp., Armonk, NY, USA). Continuous variables are presented as mean ± standard deviation (SD). Differences between pre- and post-release kinematic parameters were evaluated using paired-samples t-tests. A p-value of <0.05 was considered statistically significant. Due to the pilot nature of this study and the fixed availability of specimens, an a priori power analysis was not performed.

## Results

Surgical and visual assessment

All six cadaveric specimens underwent the complete testing protocol. Complete transection of the TCL was visually confirmed in all cases. During the simulated maximal FCU traction maneuvers post-release, no gross subluxation, dislocation, or mechanical snapping of the PT joint was observed in any of the specimens.

Kinematic measurements

A comparison of the kinematic parameters before and after TCL release revealed no significant differences. The mean difference in wrist extension was -2.83° (SD: 6.56; 95% CI: -9.72° to 4.05°; p = 0.34). The mean difference in wrist flexion under FCU loading was -2.84° (SD: 7.36; 95% CI: -10.57° to 4.88°; p = 0.39).

PT angular analysis

The primary outcome measure, the PT angle relative to the ulnar-metacarpal axis, showed a mean difference of -0.01° between the pre- and post-release states (SD: 2.70; 95% CI: -2.84° to 2.82°; p = 0.99). The complete set of quantitative data is presented in Table 1.

**Table 1:** Comparison of Kinematic Parameters and PT Angulation Before and After TCL Release Data represent mean differences (M), standard deviations (SD), and 95% confidence intervals (CI) for paired t-tests (N = 6). No statistically significant differences were observed in extension, flexion, or joint angle (p > 0.05). PT, pisotriquetral; TCL, transverse carpal ligament

Measurement	Mean Difference (M)	SD	t	p	95% CI
Wrist Extension	-2.83	6.56	1.06	0.34	-9.72, 4.05
Wrist Flexion	-2.84	7.36	-0.95	0.39	-10.57, 4.88
Pisotriquetral Angle	-0.01	2.70	-0.01	0.99	-2.84, 2.82

## Discussion

Interpretation of findings

The primary objective of this pilot study was to determine whether routine transection of the TCL compromises the immediate biomechanical stability of the PT. Our results indicate that complete TCL release does not produce statistically significant alterations in pisiform tracking or global wrist kinematics in an embalmed cadaveric model. Even under simulated maximal FCU loading, the PT angle remained constant. This suggests that, although the TCL is integral to the carpal arch, it is likely not the primary stabilizer of the pisiform. Instead, the stability of the PT joint is presumably preserved by a robust, distinct ligamentous complex - specifically, the pisohamate and pisometacarpal ligaments - which remain intact during standard CTR [9,17].

Comparison with previous literature

Our results align with biomechanical studies suggesting that the pisiform functions effectively as a sessile sesamoid with intrinsic stability, independent of the TCL [15,18,19]. However, these findings contrast with reports implicating the TCL as a critical tether for ulnar carpal kinetic [8,16]. This discrepancy is likely attributable to differences in specimen preservation; whereas fresh-frozen tissues used in other studies allow for physiological laxity, the embalmed specimens in our cohort exhibited inherent stiffness that may mask minor instability patterns [20,21]. Nevertheless, our data support the anatomical view that the primary stabilization of the pisiform is derived from the PT ligament complex rather than the flexor retinaculum itself [19,22].

Clinical implications

These biomechanical findings have immediate relevance for managing ulnar-sided wrist pain following CTR. The persistence of PT stability in our model challenges the hypothesis that iatrogenic destabilization is the primary cause of postoperative pain [6,7,18]. Consequently, clinicians should consider alternative etiologies for such symptoms, particularly the "unmasking" of pre-existing, subclinical PT arthritis [6,18]. Since the joint mechanics themselves appear unaltered by surgery, pain may arise from increased loading on degenerate cartilage rather than kinematic failure [9]. Therefore, thorough preoperative evaluation, including a PT grind test and targeted radiographic screening, is recommended to identify patients at risk for persistent ulnar pain [6,7].

Limitations

The conclusions of this study must be interpreted within the context of several limitations inherent to its design as a pilot investigation. First, the use of embalmed cadaveric tissue is a significant confounder. Formaldehyde fixation increases soft tissue stiffness, which may have artificially enhanced the stability of the PT joint compared to the more compliant soft tissues found in vivo [21,23]. Second, the sample size (N = 6) was limited by specimen availability and strict exclusion criteria regarding joint ankylosis. While sufficient for a pilot analysis, this small cohort lacks the statistical power to detect minor effect sizes (Type II error). Third, the loading protocol relied on manual traction of the FCU tendon. While we attempted to standardize the force by pulling to maximal excursion, the lack of an instrumented actuator introduces potential variability in the load applied across trials. Finally, the 2D video analysis provides a simplified vector representation of a complex 3D motion; rotational malalignment or subtle subluxations out of the camera plane could not be quantified.

Future directions

To validate these preliminary findings, future research should utilize fresh-frozen cadaveric specimens to better approximate physiological tissue properties. Furthermore, the use of 3D motion capture technology and instrumented loading rigs would provide a more granular understanding of the subtle kinematic relationships within the PT complex after surgery.

## Conclusions

In this cadaveric pilot study, complete release of the TCL did not result in immediate kinematic destabilization of the PT joint. Our findings suggest that the intrinsic ligamentous stabilizers of the pisiform are sufficient to maintain joint congruity, even in the absence of the flexor retinaculum tethering effect. Consequently, the onset or persistence of ulnar-sided wrist pain following CTR is less likely to be caused by iatrogenic instability and more likely attributable to the unmasking of pre-existing PT pathology. While these results are encouraging for the safety of standard release procedures, they must be interpreted as preliminary due to the use of embalmed tissue and limited sample size. We recommend that surgeons maintain a high index of suspicion for PT osteoarthritis in patients presenting with atypical ulnar wrist symptoms and incorporate specific preoperative evaluation of this joint to optimize the surgical outcomes.
